# Modelling the effect of spatially variable soil properties on the distribution of weeds

**DOI:** 10.1016/j.ecolmodel.2018.11.002

**Published:** 2019-03-24

**Authors:** H. Metcalfe, A.E. Milne, K. Coleman, A.J. Murdoch, J. Storkey

**Affiliations:** a Sustainable Agricultural Sciences, Rothamsted Research, Harpenden, Hertfordshire AL5 2JQ, UK; b School of Agriculture, Policy and Development, University of Reading, Earley Gate, PO Box 237, Reading RG6 6AR, UK

**Keywords:** *Alopecurus myosuroides*, Life-cycle, Soil properties, Spatial model, Patch

## Abstract

The patch spraying of weeds is an area of precision agriculture that has had limited uptake. This is in part due to the perceived risks associated with not controlling individual weeds. Nevertheless, the inherent patchiness of weeds makes them ideal targets for site-specific management. We propose using a mechanistic model to identify areas of a field vulnerable to invasion by weeds, allowing the creation of treatment maps that are risk averse. We developed a spatially-explicit mechanistic model of the life-cycle of *Alopecurus myosuroides*, a particularly problematic weed of cereal crops in the UK. In the model, soil conditions which vary across the field, affect the life-cycle of *A. myosuroides*. The model was validated using data on the within-field distribution of *A. myosuroides* on commercial farms and its co-location with soil properties. We demonstrate the important role played by soil properties in determining the within-field distribution of *A. myosuroides*. We also show that scale-dependent correlations between *A. myosuroides* and soil properties observed in the field are an emergent property of the modelled dynamics of the *A. myosuroides* life-cycle. Our model could therefore support effective site-specific management of *A. myosuroides* within fields by predicting areas that are vulnerable to *A. myosuroides*. The usefulness of this model in its ability to predict patch locations for *A. myosuroides* highlights the possibility of using similar models for other species where data are available on the response of the species to various soil properties.

## Highlights

• Incremental changes to the life-cycle of *A. myosuroides* due to soil properties, when combined in a modelling approach, reveals them as important determinants of the within-field distribution of this species.
• Scale-dependent correlations between *A. myosuroides* and soil properties observed in the field are an emergent property of the modelled dynamics of the *A. myosuroides* life-cycle.
• Our model predicts areas of field that are vulnerable to *A. myosuroides* based on soil properties and so could support effective site-specific management of *A. myosuroides* within field.

## 1 Introduction

Precision agriculture is already commonplace in many aspects of farming. Information-based management systems to adapt fertilizer distribution across the field were first introduced in the mid-1980s (Gebbers and Adamchuk, 2010) and since then precision farming techniques including GPS steering, soil mapping, and variable rate seeding are becoming increasingly popular; with the proportion of UK farmers who implement these techniques increasing over recent years (Defra, 2013). The concept of site-specific weed management, specifically patch spraying is less prevalent but is gathering interest. Site-specific weed management takes into account the spatial variability of weeds either through intermittent spraying based on observed weed density at different locations or by modelling the thresholds for weed density above which it is economic to spray (Garibay et al., 2001). This results in reduced chemical cost and more accurate application of control practices (Dieleman et al., 2000).

Despite the economic and environmental benefits of patch spraying for weed management, it has not been readily taken up as a standard management tool. There are many reasons for this (Christensen et al., 2009) but perhaps the most important is that a change to patch spraying goes against current practice. An unwillingness to implement site-specific weed management may stem from the perceived risk of missing individuals that grow outside of currently established patches and are not detected by weed mapping. Individuals may enter the field from elsewhere or a patch may expand due to increased dispersal from highly dense patches or through cultivation. If these individuals remain unsprayed there is a risk they will turn into new patches. The incorporation of buffer zones into spray maps is a general measure taken to try and combat this, however this does not account for seed spreading outside of the immediate area surrounding the patch or entering the field from elsewhere.

Despite the reservations of farmers in using precision agriculture techniques in weed control, many weed species lend themselves well to site-specific control due to their patchy distributions within fields. In recent years, the idea of studying the spatial distribution of weeds with the intent to introduce a site-specific aspect to their management has been an area of growing interest. The introduction of satellite spatial technology in the 1990s introduced the possibility of locating weed patches in the field (Lutman et al., 2002) and this is now being explored further with the use of unmanned aerial vehicles (e.g. Castaldi et al., 2016, López-Granados et al., 2016a, López-Granados et al., 2016b, Pérez-Ortiz et al., 2016). Research on the real-time mapping of weeds (e.g. Murdoch et al., 2010, Tian et al., 2000) is also ongoing.

We focus here on *Alopecurus myosuroides* Huds. (black-grass), a common grass weed of winter cereals in north-west Europe (Holm et al., 1997). It is particularly problematic due to its fast reproductive rate, strong competitive ability with the crop, and because its life-cycle is largely synchronised with that of winter cereals (Maréchal et al., 2012). *Alopecurus myosuroides* plants can produce large amounts of seeds (Moss, 1980) meaning small failures in control can lead to rapid population growth and dense infestations within fields. Currently, the main means by which farmers choose to control this pernicious weed is through broadcast application of herbicides. However, the decreasing number of chemical products available for use, and increasing economic and environmental pressures to reduce herbicide use puts a growing emphasis on the optimisation of current techniques and finding alternative approaches (Grundy, 2003). The within-field distribution of *A. myosuroides* is patchy (Wilson and Brain, 1991, Krohmann et al., 2006, Metcalfe et al., 2016, Metcalfe et al., 2018b) and as such this presents an opportunity for site-specific management.

It has been shown that the patchy distribution of *A. myosuroides* in fields can be related to variation in soil properties (Holm et al., 1997, Lutman et al., 2002, Murdoch et al., 2014, Metcalfe et al., 2016, Metcalfe et al., 2018b), particularly soil organic matter, pH, and water. Metcalfe et al. (2018b) demonstrated that these relationships are strongest at coarse scales (>20 m), making them ideal for the implementation of precision management. This provides a basis upon which to identify weed vulnerable zones within fields for this species. However, a biological model that elucidates the underlying processes and causality of these relationships could potentially be used to assess the relative propensity of other species to form patches based on their ecophysiology.

Moss (1990) constructed a basic life-cycle model of *A. myosuroides*. Colbach et al., 2006, Colbach et al., 2007 added more complexity to their life-cycle model for *A. myosuroides*: ALOMYSYS. Most weed population dynamics models ignore the within-field distribution of the weed and generally simulate the average density (Holst et al., 2007). Paice et al. (1998), however, considered the need for a spatial component to models of *A. myosuroides* population dynamics. Building on basic models of the *A. myosuroides* life-cycle they incorporated elements of stochasticity into the life-cycle processes and included a binomial probability of weed survival following herbicide application. They included both isotropic and anisotropic dispersal processes derived from Howard et al. (1991). They showed that when dispersal only occurs over short distances, patchiness is maintained and even if the field is initialised with a uniform seed bank the population will develop patches in a homogeneous environment. Gonzalez-Andujar et al. (1999) also considered spatial patterns in the modelling of *A. myosuroides* in an array of hexagons representing part of a field. Seed dispersal was assumed to be isotropic and dispersal by the combine was also considered. The inclusion of features such as seed dispersal in spatial models are often not sufficient to describe the degree of patchiness observed in field populations. It is thought that this may be due to the omission of the effect of soil variables (Paice et al., 1998, Rew and Cousens, 2001).

Dunker et al. (2002) included the effect of nutrients, soil pH and particle size on *A. myosuroides* in their model, based on the results of a pot experiment. They verified this model in one field where *A. myosuroides* counts and soil properties were measured on a 50 m × 50 m grid. Their model was based on the demographic data from Moss (1990). In the Dunker et al. (2002) model, only the early parts of the life-cycle are affected by soil as the experiment on which they based their model was only conducted for 5 weeks post germination. They found their simulations to be only weakly correlated with real data and only 4 out of 20 simulations showed a significant correlation.

Our aim was to develop a model to help address the risk, associated with current patch spraying techniques, of missing individuals that disperse outside of currently established patches. We propose an extension to current techniques for mapping weeds, which addresses this concern: that is to identify parts of the field that are vulnerable to weed infestation. These “weed vulnerable zones” can then be used to guide the precision application of herbicides. To do this, we develop a spatially explicit model of the life-cycle model of *A. myosuroides* incorporating mechanistic responses to soil variability across the whole life-cycle. The model is based on the work of Moss (1990), Colbach et al. (2006) and Paice et al. (1998) but extended to include the direct and indirect effect of soil on the weed based on experimental data. It also introduces stochasticity into the system providing a more realistic range of possible outcomes than could a deterministic model. By modifying the life-cycle of the plant according to known responses to variation in soil properties (including texture, organic matter, water and pH) we tested the hypothesis that scale-dependent relationships between soil properties (including soil organic matter, water content and pH) and the density of *A. myosuroides* observed in fields by Metcalfe et al. (2018b) can be modelled. Our model strikes a balance between tractability and complexity—processes are only modelled mechanistically to capture the effect of different soil properties. It was built to answer the specific hypothesis that the heterogeneous environment impacts on population dynamics resulting in scale-dependent correlations between soil properties and *A. myosuroides* distributions and does not claim to predict absolute numbers but rather the relative fitness in different within-field locations.

## 2 Model description

We developed a spatially explicit model of *A. myosuroides* population densities within a field, incorporating various processes throughout the plant's life-cycle. The model is described here and the parameterisation detailed in the following section. The modelled field is described by a grid of square cells, the side length of which can be defined in real units of distance. We define the relative position of these cells in Cartesian coordinates and so a rectangular area of defined size can be simulated allowing spatial processes, such as dispersal, between cells. For each grid cell we define values for soil texture (% clay and silt), soil pH, soil organic matter (%), soil gravimetric water content (%), slope, and aspect, at a resolution consistent with the chosen grid size.

### 2.1 Soil water content

Soil water content is dynamic and changes over time according to weather and other soil properties. Plant available water, given as soil volumetric water content (*S*_VWC_) is calculated by

(1) $$S_{\mathrm{VWC}}=\frac{S_{\mathrm{GWC}}\times D_{b}}{100}.$$

where *S*_GWC_ is the soil gravimetric water content set for the cell and *D*_*b*_ is the bulk density of the soil, which is calculated using the pedotransfer function:

(2) $$\begin{array}{ccc} D_{b} & =0.80806+0.823844\exp\left(-0.27993\;S_{\mathrm{SOM}}\right)+0.0014065\left(100-S_{\mathrm{Clay}}-S_{\mathrm{Silt}}\right)-0.0010299\;S_{\mathrm{Clay}} & \; \end{array}$$

derived by Hollis et al. (2012) for cultivated topsoil, where *S*_SOM_ is the soil organic matter (%), *S*_Clay_ is the soil clay content (%) and *S*_Silt_ is the soil silt content (%).

We modelled the change in volumetric water content of the soil on a daily time step with additions from daily precipitation and losses from evapotranspiration. Evapotranspiration was calculated for a bare soil surface in the autumn, and a crop canopy at other times of the year. For these calculations we followed the analysis by Penman (Frere and Popov, 1979, Penman, 1948, Penman, 1956, Penman, 1963). The Penman formulae are dependent on the evaporative demand of the atmosphere and the net absorbed radiation, which are calculated using daily temperature, solar irradiation, vapour pressure and wind speed. Other required values including reference values for albedo and constants used in the formulae were taken from FAO guidelines for computing crop water requirements (Allen et al., 1998).

We modify the solar irradiation from the input weather data according to topography (shown to be an important determinant of *A. myosuroides* patch location by Metcalfe et al., 2018b), before using it in the Penman calculations. We split daily irradiation into its direct and diffuse component parts according to the latitude (Kropff, 1993, Kropff et al., 1993). Each cell, irrespective of topography, received the full amount of diffuse irradiation, but the direct component was modified according to the slope and aspect of the field in each cell by scaling it up or down relative to a reference value for a flat field so that steep south facing slopes received more direct radiation than shallow or north facing slopes (Frank and Lee, 1966).

As water potential of soil varies with soil type, we calculate this using the van Genuchten pedotransfer function (van Genuchten, 1980; as parameterised by Wösten et al., 1999), which allows conversion between volumetric water content and water potential according to the soil clay, silt, and organic matter content and the bulk density of the soil. If the water potential exceeds 15,000 mbar then we assume the grid cell has reached wilting point and no more water can be lost. Conversely if the water potential drops below 50 mbar then the field is at capacity and any additional water input will drain through and so the water content of the soil does not increase (Wösten et al., 1999).

### 2.2 *A. myosuroides* life-cycle

**Fig. 1:**
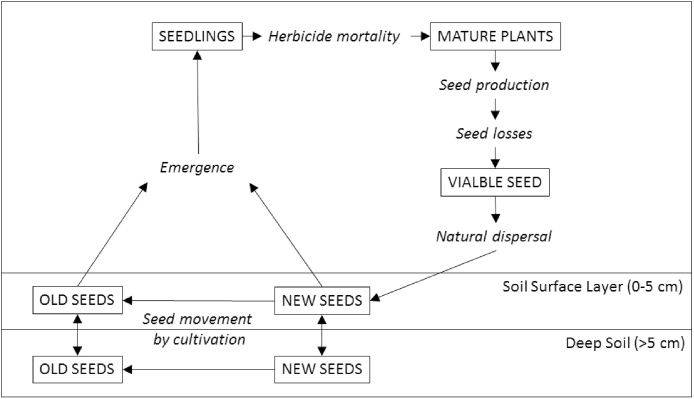
Basic component structure of the *A. myosuroides* life-cycle model. Processes are shown in italics and components of the *A. myosuroides* life-cycle are boxed and capitalised. This life-cycle component is based on the model by Moss (1990) and runs in each cell of our spatially explicit model. Note: *A. myosuroides* seed is not spread by the combine while still on plant and so movements by cultivation only occur in the soil. This may not necessarily be the case for other weed species that retain seed heads until harvest.

The life-cycle of *A. myosuroides* is modelled in 4 main stages: seedlings, mature plants, viable seed, and the seedbank, as described by Moss (1990). In each iteration of the model there are two cohorts of seeds in each of two soil layers; new seeds (shed in the previous year) and old seeds (shed in any year prior). Each stage of the life-cycle is connected to the next by one or more processes (see Fig. 1). The life-cycle runs independently in each grid cell with various processes being affected by the soil properties associated with that cell. We identified the key transitions in the life-cycle that we expected to respond to variability in soil properties (Table 1). We included additional functions (described below) in the model to predict the effects of variable soil properties on the whole life-cycle.

**Table 1:** Stages of the *A. myosuroides* life-cycle identified as being affected by soil variability.

Life-cycle stage	Soil property	Effect	Source
Germination	Plant available water	Total germination increases with hydrothermal time	Colbach et al. (2006) and supplementary pot experiments
Germination	Soil pH	More seeds germinate at low pH	Metcalfe et al. (2017)
Pre-emergence herbicide mortality	Soil organic matter	Probability of survival increases with increasing soil organic matter	Metcalfe et al. (2018a)
Seed production	Soil organic matter	Seed production increases with soil organic matter	Supplementary pot experiments
Seed production	Water stress	Seed production decreases when water is limiting	Storkey and Cussans (2007)

#### 2.2.1 *A. myosuroides* emergence

We calculate the number of *A. myosuroides* seedlings that emerge by taking the number of seeds in the soil surface layer and multiplying this by the proportion of seeds germinating (*G*). We model the proportion of seeds that germinate (*G*) as a function of hydrothermal time (*θ*_HT_) following Colbach et al. (2006):

(3) G=M[1−e(−k(θHT−ta−a)x50−a)c]when   x>a+taG=0otherwise

where *M* is the maximum level of germination, *a* is the lag phase of germination, *c* is a shape parameter, and *x*_50_ is the time to 50% germination. These parameters are modelled according to properties relating to the seeds (see supplementary material for calculations) including the age of the seed which allows different values of *G* for the old and new seed cohorts, time from germination to maturity (Julian day of maturity drawn from a normal distribution) of the mother plants, water deficit between flowering (Julian day of flowering drawn from a normal distribution) and maturity, depth of seed, hydrothermal time spent in darkness prior to tillage, mean seed mass, and total available nitrogen. The offset *t*_*a*_ increases the delay before the commencement of germination. We calculate the water deficit experienced by the parent plants from the previous flowering to previous harvest by taking the difference between the daily evapotranspiration and the sum of the soil water content and daily precipitation. If more water is lost to evapotranspiration than is available then this difference is added on to the water deficit.

Hydrothermal time (*θ*_HT_) is accumulated on a daily timestep from the day of tillage (Julian day of tillage drawn from a normal distribution) for a maximum of 50 days by

(4) $$\theta_{\mathrm{HT}}=\theta_{H}\theta_{T}\;\mathrm{where}$$

(5) $$\begin{array}{cc} \theta_{H} & =\psi -\psi_{b}\;\mathrm{if}\;\psi >\psi_{b}\\ \theta_{H} & =0\;\mathrm{otherwise} \end{array}$$

and

(6) $$\begin{array}{cc} \theta_{T} & =T-T_{b}\;\mathrm{if}\;T>T_{b}\\ \theta_{T} & =0\;\mathrm{otherwise} \end{array}$$

*ψ* is the daily water potential (MPa) and *T* is the daily temperature (°C). *ψ*_*b*_ and *T*_*b*_ are the base water potential and temperatures required for germination respectively.

Crop germination is much less variable than that of the weed due to their larger seeds and breeding efforts toward uniform establishment (Stratonovitch et al., 2012) and so is modelled by thermal time (it is assumed to be unaffected by the soil) using a function from Storkey and Cussans (2000) which divides the crop green area (*A*_*G*_, Eq. (7)) by the area of the cell. If the crop green area index reaches 0.5 before day 50 then *A. myosuroides* germination is terminated at that point.

(7) $$A_{G}=\frac{(C_{m}/G_{m})\;\ln\left\{1+\exp\left[G_{m}(t_{\mathrm{sum}}-t_{0})\right]\right\}}{10,000}$$

where *C*_*m*_ is a maximum growth rate (g plant^−1^ °d^−1^), *G*_*m*_ is a maximum relative growth rate (°d^−1^), *t*_sum_ is the accumulated time from the day of tillage, and *t*_0_ is the time at which the plant effectively reaches a linear phase of growth (°d).

#### 2.2.2 Herbicide mortality

The number of plants surviving herbicide application is drawn from a binomial distribution:

(8) $$P(i|t,p)=\left|\left(\begin{array}{c} t\\ i \end{array}\right)\right|p^{i}{\left(1-p\right)}^{t-i}$$

where *P*(*i*) is the probability of *i* plants surviving from an initial number of *t* plants in the cell, given a probability, *p* of survival.

Pre-emergence herbicide efficacy is known to be reduced with increasing organic matter, this can have an indirect effect on the life-cycle of the weed as on some soils there will be an increased number of survivors (Pederson et al., 1995, Metcalfe et al., 2018a). So to model the proportion of plants surviving pre-emergence herbicide application the value of *p* used to draw from the binomial distribution (Eq. (8)) is modelled by:

(9) $$p=\frac{\eta \;S_{\mathrm{SOM}}}{1+\zeta \;S_{\mathrm{SOM}}}+\tau ,$$

where *S*_SOM_ is the percentage soil organic matter.

#### 2.2.3 Seed production

Seed head production is density dependent and so we model the number of heads per cell (*D*_Heads_) by

(10) $$D_{\mathrm{Heads}}=\frac{\beta \;D_{\mathrm{Plants}}}{1+\alpha \;D_{\mathrm{Plants}}}\cdot T_{A:P}$$

where *D*_Plants_ is the number of plants per cell (Moss et al., 2010) and *T*_*A*:*P*_ is the mean ratio of actual to potential soil transpiration (given by Eq. (11)) over the growing season. This accounts for the effect of water stress on plant yield (Osakabe et al., 2014).

(11) $$T_{A:P}=\frac{1}{1+\epsilon \;\exp(\rho \times F_{\mathrm{TSW}})}$$

We calculate the fraction of transpirable soil water (*F*_TSW_) by taking the average of the daily soil volumetric water contents from the start of germination to the start of flowering as a proportion of the difference between field capacity and wilting point for that soil type.

The number of seeds produced per head is stochastic and is sampled from a log-normal distribution. A proportion of this total seed production will be non-viable and is sampled stochastically from a normal distribution.

#### 2.2.4 Seed losses

The amount of seed lost, for example from predation, is sampled from a lognormal distribution and seed survival in the soil is sampled from a normal distribution.

#### 2.2.5 Seed movement

We modelled both natural *A. myosuroides* seed dispersal and dispersal of seed by cultivation. The probability distribution for each dispersal process was calculated by numerical integration as:

(12) $$P(m,n)=\underset{S(n-0.5)}{\int^{S(n+0.5)}}\underset{S(m-0.5)}{\int^{S(m+0.5)}}f(x,y)\;\mathrm{dx}\;\mathrm{dy}$$

where *S* is the side length of the cell, *P*(*m*, *n*) is the probability of a seed falling into a cell at the distance from the source *x* = *m*, *y* = *n* and *f*(*x*, *y*) is the dispersal probability function.

**Fig. 2:**
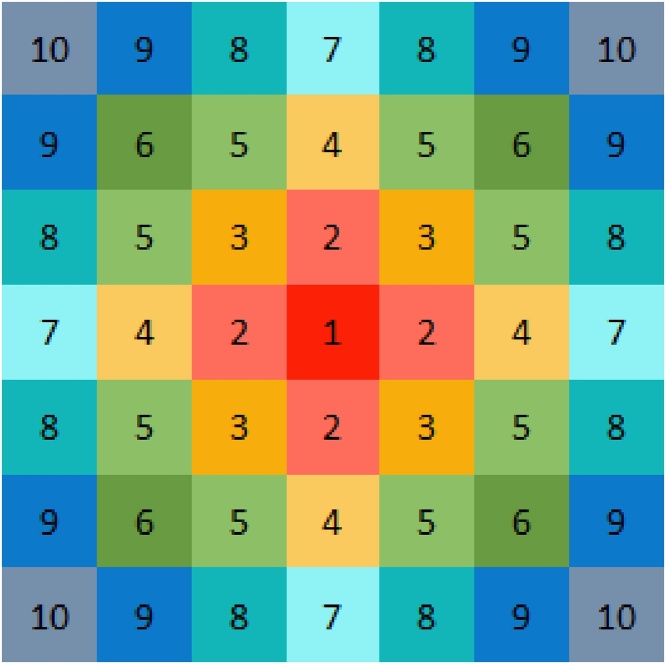
Numerical order of assessment of nearby squares for the natural dispersal of seeds from a plant in the centre square (labelled “1”). Cells with the same number all receive the same proportion of seed from the starting cell. If required, the pattern continues in the same manner expanding outwards.

*Natural dispersal*. The natural dispersal of *A. myosuroides* seed is assumed to be isotropic and to follow the rotated Gaussian distribution

(13) $$f(x,y)=\frac{1}{2\pi \sigma^{2}}\exp\left[-0.5\left[{\left(\frac{x-\mu }{\sigma }\right)}^{2}+{\left(\frac{y-\mu }{\sigma }\right)}^{2}\right]\right]$$

(Paice et al., 1998). The probability of seed falling into each cell is calculated in turn by Eq. (12) following the order indicated in Fig. 2 until a total proportion of 0.999 has been accounted for. If any seeds remain these are dispersed to a randomly allocated cell to represent other sources of seed dispersal not accounted for here. The resulting list of proportions are stored and used throughout each yearly cycle of the model to move seeds from one cell to nearby cells.

*Seed movement by cultivation*. Dispersal by cultivation is anisotropic with seeds being dispersed in the direction of travel (Lutman et al., 2002). In order to model the way in which seeds were moved by the combine, we assumed

(14) $$f(x,y)=\frac{\gamma }{2}\;\exp^{\frac{\gamma }{2}\left(2\varepsilon +\gamma \lambda^{2}-2x\right)}\mathrm{erfc}\left(\frac{\varepsilon +\gamma \lambda^{2}-x}{\sqrt{2\lambda }}\right)$$

This distribution matches the shape of that described by Paice et al. (1998) for anisotropic dispersal of seeds. The distribution is integrated for each grid cell in turn for a maximum of five grid cells in the direction opposite to the direction of travel and then towards the direction of travel until a total proportion of 0.999 is accounted for. Any remaining seeds are randomly allocated to a grid cell in the direction of travel, up to a maximum dispersal distance of 20 m. The direction of travel is set up along the *x* axis of the grid from west to east for the first set of rows up to the width of the cultivator (40 m). It is then switched to travel east to west. The direction changes every time the number of rows reaches a multiple of the cultivator width.

For both natural dispersal and the seed movement by cultivation, if seeds are to be moved into a cell that lies outside of the model arena the process is reflected such that there is no immigration or emigration of seeds to and from the field. This is representative of a real field where boundaries are a source of seed (Marshall, 1989).

*Vertical movement of seed in the soil*. Seeds are moved vertically between the shallow and deep soil layers. The tillage type can be set for each year to one of 4 types: plough, 20 cm tine, 10 cm tine, or <5 cm tine. In years when the tillage is “plough” a proportion of seeds from the shallow soil layer are buried into the deep soil layer, conversely some seeds are brought up to the shallow soil layer. For all other tillage types there is no upward movement of seed (from the deep soil layer to the shallow soil layer) but a proportion of seeds are buried according to the depth of tillage.

## 3 Model parameterisation

Where possible, each function in the life-cycle model was parameterised using data from the literature. However, the effect of soil organic matter (and its influence on water holding capacity) on *A. myosuroides* emergence and seed production was identified as a knowledge gap and so we set up an experiment under controlled conditions in order to parameterise these functions within the model (see supplementary material for experimental details).

### 3.1 *A. myosuroides* emergence

**Fig. 3:**
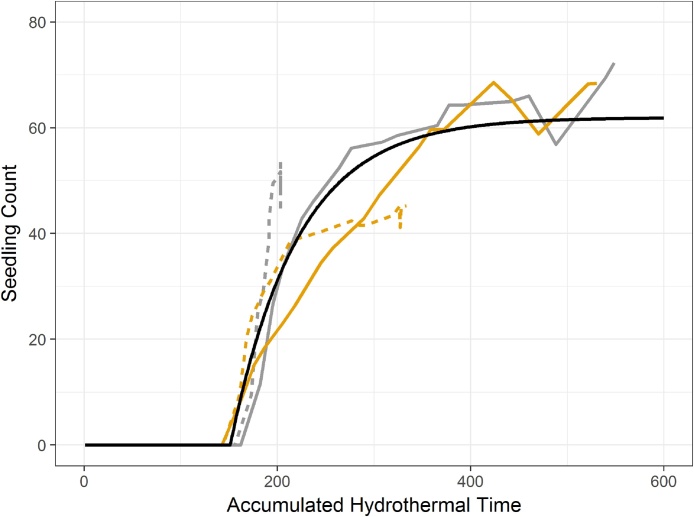
Germination counts plotted against hydrothermal time. Grey is low soil organic matter, Yellow is medium soil organic matter. Solid lines show high water input and dashed lines are low water input. The solid black line shows the resulting germination counts from Eq. (3) when parameterised for the seeds used in the experiment. See supplementary material for experimental detail.

Colbach et al.'s parameterisation of Eq. (3) (2006, see supplementary material) relies on information about the germinating seeds including the age and seed mass. To determine whether this parameterisation works on soils with very different water holding capacities we germinated seeds of known origin, age and size on soil with different levels of organic matter (see supplementary material). The data from this experiment supported the shape of the Colbach curve but the offset parameter *t*_*a*_ is increased to 49.169 in our fit (Fig. 3). This offset increased the delay before the commencemenrt of germination to match the mean of our four treatments (Fig. 3).

Germination counts on different levels of soil pH collected by Metcalfe et al. (2017) show that the asymptote for germination is higher when soil pH is low. We therefore included a pH threshold of 6.5 in our model. At pH levels below this threshold *M* (Eq. (3)) is increased by the ratio of the values for the asymptote of the curves fitted to the low and high pH treatments respectively (40.92/36.72) (Metcalfe et al., 2017).

In the parameterisation of Eq. (3) (and Eqs. (18)–(26) in the supplementary materials) we set the age of the old cohort of seeds to 818 days and the new cohort to 60 days. Changing this parameter allowed the germination of seeds to occur at different rates. The mean Julian day of tillage, flowering of *A. myosuroides* and maturity of *A. myosuroides* were set to be 258, 150, and 206 with respective variances 8, 3, and 6. These were determined using field data from a series of field experiments reported in Storkey and Cussans (2007) on *A. myosuroides* competition in winter wheat sown in the autumn and managed according to standard farm protocols except for the absence of herbicide. Depth was set to 1.5 cm and mean seed mass, determined by weighing 100 seeds, was 0.0014 g. Total available nitrogen was set at 25 kg/ha.

Following Colbach et al., 2002a, Colbach et al., 2002b, the base temperature (*T*_*b*_) and water potential *ψ*_*b*_ for *A. myosuroides* (Eqs. (4), (5), (6)) were set at 0°C and −1.53 MPa respectively.

### 3.2 Herbicide mortality

We modelled the survival rate of *A. myosuroides* after the application of pre-emergence herbicides using data from Metcalfe et al. (2018a). We took the data points for the proportion of seedlings surviving an application of pre-emergence herbicide at a dose equivalent to field rate on different soils and plotted this against the organic matter (%) in the soil and fitted Eq. (9) to the data. The fitted values were *η* = 4.9, *ζ* = 3.8252, and *τ* = −1.0890 (see supplementary Figure S.3).

For post-emergence herbicide application we assume no effect of soil variability on contact herbicide efficacy and so *p* was fixed at 0.3 (Bayer CropScience, 2017).

### 3.3 Seed production

Moss et al. (2010) parameterised their model of density dependent head production (Eq. (10)) with data from 16 field experiments to give parameter values *β* = 8.71 and *α* = 0.005741. We adjusted this equation to account for soil organic matter according to the results from our experiment (see supplementary material). We only had one plant per pot, in our experiment, and so we would not expect this to be representative of the number of heads produced under field conditions but assumed the relative differences were representative of those seen under field conditions. This allowed us to compute a generic relationships between soil organic matter and the density dependent relationship between plants and heads (see supplementary material for derivation).

Parameters in Eq. (11) were derived for *A. myosuroides* as *ϵ* = 6.88 and *ρ* = −4.61 from a series of glasshouse experiments reported in Storkey and Cussans (2007).

The mean (4.58) and standard deviation (0.23) of the lognormal distribution used to determine seed production per head are estimated from data provided by Moss (1990). The normal distribution: N(0.55,0.126) is used to draw values for the proportion of viable seed, the mean and standard deviation are again estimated from the data provided by Moss (1990).

### 3.4 Seed losses

For the distributions used to calculate seed losses, data from Moss (1990) were again used to estimate the means and standard deviations. The distributions used were: Log-normal(−0.81, 0.13) for above ground seed losses (e.g. through predation), and N(0.3,0.077) for seed survival in the soil.

### 3.5 Seed movement

As was described by Paice et al. (1998), the mean (*μ*) of the natural dispersal distribution (Eq. (13)) is set at 0 and the standard deviation (*σ*) at 0.3. For seed movement by cultivation (Eq. (14)) parameters were set to *γ* = 10/3, *λ* = 0.1 and *ε* = −0.15 to best match the shape of the distribution described by Paice et al. (1998) for anisotropic dispersal of seeds. The proportion of seeds moved vertically in the soil for each type of tillage was described by Moss (1990). Here stochasticity is added by drawing these from distributions estimated from the mean and range of that original data (Table 2)

**Table 2:** Distributions used to sample the proportion of seeds to be moved between soil layers due to tillage.

Tillage type	Direction of movement	Distribution type	Mean	Variance
Plough	Shallow to deep	Lognormal	−0.0515	0.0191
	Deep to shallow	Lognormal	−1.0670	0.1199
20 cm tine	Shallow to deep	Normal	0.2000	0.0510
	Deep to shallow	None		
10 cm tine	Shallow to deep	Normal	0.4000	0.1010
	Deep to shallow	None		
<5 cm tine	Shallow to deep	None		
	Deep to shallow	None		

## 4 Model validation

Metcalfe et al. (2018b) linked *A. myosuroides* seedling counts to various environmental properties within fields commercially producing winter wheat. They sampled 136 locations in each field using an unbalanced nested sampling design, with pairs of points separated by fixed distances. At each sampling point they counted the number of *A. myosuroides* seedlings emerging in the autumn. They also took soil cores to analytically determine the soil clay and silt content, soil organic matter, soil pH and soil gravimetric water content. The nested design structure allowed the partitioning of the components of variance for both *A. myosuroides* and soil properties at each of the spatial scales studied using the residual maximum likelihood (REML) estimator as described by Metcalfe et al. (2016), and scale-dependent correlations between *A. myosuroides* counts and soil properties were calculated.

### 4.1 Patch location

To validate our model, we simulated three UK fields, for which complete datasets on soil properties and weed densities were available, studied by Metcalfe et al. (2018b). The three fields were Harpenden (Hertfordshire), Redbourn (Hertfordshire) and Haversham (Buckinghamshire) (see Metcalfe et al., 2018b). As an initial investigation, we kriged the measured soil data to predict on a 1-m grid. We then used this to parameterise the cells in the model. We simulated 40 years of weed growth starting with an initial seed bed of 10,000 seeds per cell, 20% of which were in the top soil layer. As we did not know the tillage history of the studied fields, we simulated three typical tillage systems: (i) rotational cultivation with three years of tillage at <5 cm followed by one year using the plough, (ii) tillage at 10 cm, and (iii) tillage at <5 cm. We ran the model with all required input weather data on a daily timestep from Rothamsted met station (Hertfordshire, UK) beginning with data from 1966. We discarded the simulation results from the first 10 years to allow the patches to stabilise following the initial seeding. We recorded and mapped the average number of plants simulated at each location in the field (1 m × 1 m grid cell) across years 11–40 and for 10 different simulations of the model (a total of 300 realisations of the field), we then compared these maps with the kriged distribution of *A. myosuroides* plants obtained by Metcalfe et al. (2018b) for that field.

### 4.2 Scale-dependent correlations

We wanted to see if the scale-dependent relationships between *A. myosuroides* and soil properties found in the field by Metcalfe et al. (2018b) were an emergent property of the model. To do this we needed to simulate soil realistic of that found in the fields, but that maintained fine-scale variation, which is lost in the kriged maps. We simulated soil properties on a 1 m × 1 m grid using lower upper decomposition of the covariance matrix, also known as the Cholesky decomposition technique (Webster and Oliver, 2007, chapter 12). We created the covariance matrix for each soil property in each field from the covariance function corresponding to the spherical variogram fitted to the soil data and conditioned the simulation to include our measured soil properties at the location where they were measured. The *R* conditioning data were transformed to standard normal form (denoted by the vector **z**) and the values at *S* unsampled positions were drawn independently at random from a standard normal distribution (vector **g**). To obtain the vector of conditionally simulated values (**y**) we used

(15) $$\text{y}=\left[\begin{array}{cc} {\text{z}}_{R} & \\ {\text{L}}_{\mathrm{SR}}{\text{L}}_{\mathrm{SS}}^{-1}+{\text{L}}_{\mathrm{RR}}{\text{g}}_{S} & \end{array}\right]$$

where **L** is the lower triangular matrix obtained from the decomposition of the covariance matrix for the field.

Following the simulation of the soil, we scaled the simulated values to match the mean and range of the original data values:

(16) $$y=\frac{x-m}{s}\times s_{\mathrm{obs}}+m_{\mathrm{obs}}$$

where *x* is a simulated value, *m* and *s* are the mean and standard deviation respectively of all the simulated values for that soil property and *m*_obs_ and *s*_obs_ are the mean and standard deviation respectively for the observed data. Ideally we would have simulated all soil properties based on their covariances. However, due to the size of the field and the spatial scale of simulation this was not possible and so we performed a number of checks to prevent the simulation of impossible soil distributions. We checked that the scaled simulated values did not exceed realistic ranges for these soil properties and discarded any simulations falling outside of the acceptable range. We also checked that the simulated clay and silt values did not sum to values greater than 100 and so we paired simulations accordingly. We produced 35 suitable simulations for each soil property for the Harpenden and Haversham fields (the Redbourn field was too large to simulate in this way).

We simulated 40 years of weed growth starting with an initial seed bed of 10,000 seeds per cell, 20% of which were in the top soil layer. We implemented 10 cm tine as the tillage type. We ran the model with weather data from Rothamsted met station (Hertfordshire, UK) beginning with data from 1966.

We took the output of the model only for years 11–40 from each simulation and extracted the number of simulated *A. myosuroides* plants at each of the sampling locations from the original study (Metcalfe et al., 2018b). We did the same analysis of the nested sampling design as described by Metcalfe et al. (2016) to give scale-dependent correlation coefficients between the simulated *A. myosuroides* counts and each soil property present in the model (clay, organic matter, pH and water) for each of the 1050 realisations of the field. We plotted a histogram to look at the frequency of these correlations across all 1050 realisations of the field given by the model and compared this distribution to the value obtained in the field data for each spatial scale and each soil property (Metcalfe et al., 2018b).

## 5 Results

### 5.1 Patch location

**Fig. 4:**
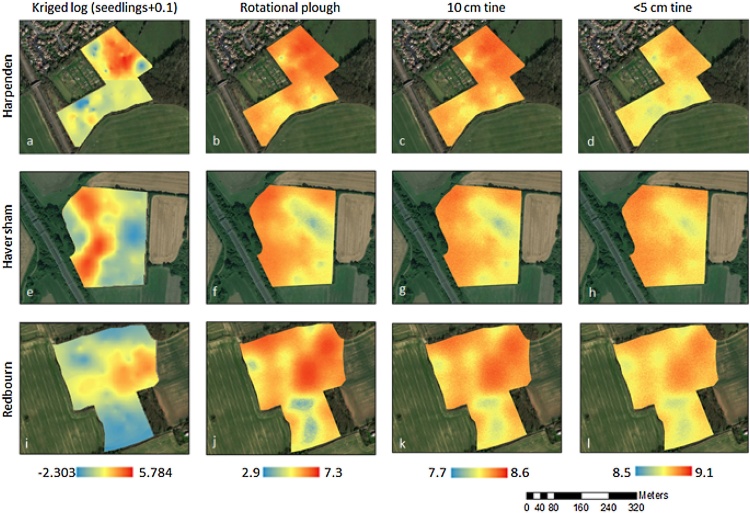
Maps of Harpenden (top row: a–d), Haversham (middle row: e–h) and Redbourn (bottom row: i–l) showing the kriged log seedling counts (first column: a, e and i) and model outputs (columns 2–4: b–d, f–h, and j–l). Each model output shows the average log seedling density in each cell across 300 realisations of the field. The simulations in the second column (b, f, and j) are the output from the model simulations with rotational ploughing as the cultivation type — ploughing every fourth year with tining at <5 cm in the intermediate years. The simulations in the third column (c, g, and k) used 10 cm tining each year, and the simulations in the fourth column (d, h, and l) used <5 cm tining. Colour scales are maintained within columns and are applicable to each cultivation type separately.

The locations of the patches predicted by the model were broadly similar to those observed in the field study (Fig. 4). At a coarse scale there are broad similarities between the distribution of *A. myosuroides* observed in the field and the predicted distributions from the model for all fields. In Harpenden, the rotational ploughing system led to very similar distributions, whereas the other two tillage systems (10 cm tine, and <5 cm tine) showed much more uniform distributions across the field (Fig. 4a–d). For the field in Redbourn the *A. myosuroides* counts in the eastern part of the field were reflected in the predictions, as were the low counts in the southern part of the field. However, in the west the observed and predicted distributions differ (Fig. 4i–l). Finally, in Haversham the western part of the field shows similar patch locations to those observed in the field (Fig. 4e–h). In all cases the predicted seedling densities are larger than were observed in the field and the patches more extensive.

### 5.2 Scale-dependent correlations

**Fig. 5:**
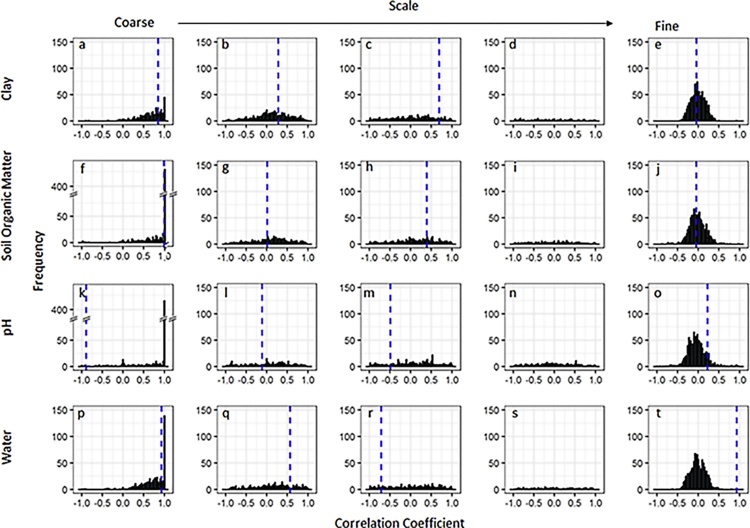
Frequency distribution of scale-dependent correlation coefficients between the simulated number of *A. myosuroides* seedlings and simulated soil properties used as inputs into the model simulations for the field in Harpenden. The dotted line represents the observed scale-dependent correlation in the field (Metcalfe et al., 2018b). The correlations shown are between *A. myosuroides* seedlings and the soil properties clay (a–e), soil organic matter (f–j), pH (k–o) and water (p–t) and for each soil property a range of spatial scales are considered ranging from coarse-scale in the first column to fine-scale in the last column: 50+ m (a, f, k, p), 20 m (b, g, l, q), 7.3 m (c, h, m, r), 2.7 m (d, i, n, s), and 1 m (e, j, o, t).

**Fig. 6:**
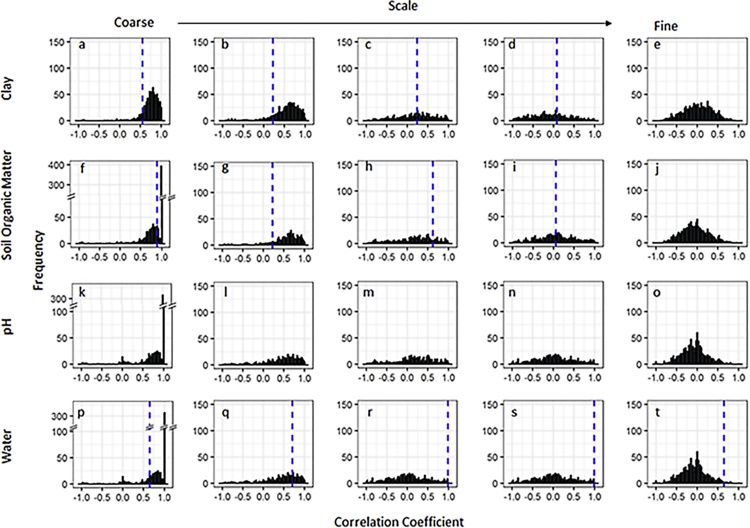
Frequency distribution of scale-dependent correlation coefficients between the simulated number of *A. myosuroides* seedlings and simulated soil properties used as inputs into the model simulations for the field in Haversham. The dotted line represents the observed scale-dependent correlation in the field (Metcalfe et al., 2018b). The correlations shown are between *A. myosuroides* seedlings and the soil properties clay (a–e), soil organic matter (f–j), pH (k–o) and water (p–t) and for each soil property a range of spatial scales are considered ranging from coarse-scale in the first column to fine-scale in the last column: 50+ m (a, f, k, p), 20 m (b, g, l, q), 7.3 m (c, h, m, r), 2.7 m (d, i, n, s), and 1 m (e, j, o, t).

The scale-dependent correlations between *A. myosuroides* and clay were fairly consistent with those observed in the field. At coarse scales the model simulations largely resulted in large positive correlations (Figs. 5a and 6 a). For Harpenden, this was close to the observed correlation in the field of 0.85 and for Haversham the simulated correlations were often larger than that observed in the field (0.55), whereas at intermediate scales (Figs. 5b–d and 6 b–d) where the observed correlation in the field were weaker the prediction from the models were less conclusive with a range of correlation coefficients that included both positive and negative values. At the finest scale all correlations between clay content and the simulated *A. myosuroides* seedling densities were small and often close to zero. This reflects the non-significant correlations observed in Harpenden and Haversham fields.

The results were similar for the relationships predicted between soil organic matter and *A. myosuroides* seedling densities with the model predicting large positive relationships with organic matter at coarse scales (Figs. 5f and 6 f). There was no distinct pattern in the correlation coefficients at intermediate scales in Harpenden (Fig. 5g–i) and only small positive correlations at intermediate scales in Haversham (Fig. 6g and h), which were similar to the observed correlations of 0.22 and 0.62 at those scales. In both fields there were correlation coefficients close to zero at the finest scale between soil organic matter and *A. myosuroides* (Figs. 5j and 6 j).

When we consider pH and its relationship with *A. myosuroides* seedling densities in the Harpenden field we find a large peak of positive correlation coefficients at coarse scales (Fig. 5k), which is in contrast to the negative correlation coefficient observed int he field. Again, at intermediate scales (Fig. 5l–n) there is no distinct pattern in the correlation coefficients and at fine scales all correlation coefficients are close to zero. In Haversham the REML model used to partition the variance across scales could not be fitted to the field data and so no comparison can be made with the simulated model outputs.

We found positive relationships with soil moisture content at the coarse-scale in the majority of simulations (Figs. 5p and 6 p). This result matched the significant positive correlation we found in the fields at this spatial scale. At intermediate scales (Figs. 5q–s and 6 q–s), the correlations between soil water content and *A. myosuroides* densities predicted by the model were less consistent. At the finest scale (Figs. 5t and 6 t) the relationship between soil water content and *A. myosuroides* seedling counts predicted by the model was often close to zero in both fields. However, at this fine scale our field observations gave quite large correlations and lay outside of the distribution of correlations predicted by our model.

## 6 Discussion

Our results support in-field studies (Lutman et al., 2002, Murdoch et al., 2014, Metcalfe et al., 2018b) that show soil is an important determinant in the within-field distribution of *A. myosuroides*. Our results suggest that our model can provide a good prediction of the location of patches within fields. Irrespective of the tillage type implemented in the model the spatial distribution of *A. myosuroides* seedlings across 300 realisations was consistent with observed field distributions (Fig. 4). This indicates the usefulness of this model in locating *A. myosuroides* vulnerable zones within fields.

Simulated seedling densities were quite different under the different tillage types, yet all provided a good estimation of patch location. This supports the conclusions from Colbach et al. (2000) that densities are often highly variable and so the prediction of densities is less accurate than the prediction of patch location. This means that it is possible to predict patch locations or weed vulnerable zones irrespective of the tillage practices in place on a farm, making the model useful as a decision support tool as it is not necessary to provide all the information about cultivation history in order to locate weed vulnerable zones. Our model was built to answer the question of the impact of soil variation on the distribution of the weed and so relative abundances are a useful output, and absolute values are not particularly important.

Strong coarse-scale relationships between soil properties and *A. myosuroides* distributions are an emergent property of our model. These matched those observed in-field. This is important as it is at these coarse scales that in-field correlations are strongest (Metcalfe et al., 2016, Metcalfe et al., 2018b) and so it is important that our model corroborates these observations. In the application of site-specific weed management most farm machinery operates at coarse scales. As such, if we can input pre-existing or supplemented soil maps, already in use on farm for other site-specific management practices, then we should be able to predict the likelihood of parts of the field being vulnerable to *A. myosuroides* and so be able to develop maps for patch spraying based on the output of this model.

As with all models of weed population dynamics there are some limitations to this model including a lack of field data, and the adoption of number of assumptions. However these are necessary in order to keep the model simple enough to be functional whilst retaining enough detail to understand the system (Fernandez-Quintanilla, 1988). Initially some of the limitations of the model come due to a lack of field data and are also under-represented in other models of *A. myosuroides* such as those by Moss (1990) and Colbach et al. (2006). These include the fate of seeds after shedding, where we have included a certain amount of seed loss but this is an all encompassing figure, including predation and decay, which would be difficult to model mechanistically. Similarly for other life-cycle processes where we only have information on the range and mean of field data such as seed production. In these cases we draw values stochastically but the chosen values remain unaffected by other processes within the life-cycle. In our model we assume that the density of the crop and other weeds are uniform across the field and so spatial variability in interspecific competition is excluded. As we base this model on the premise that the field is a heterogeneous environment this may not be a correct assumption to make, however, it is an assumption that is also made in other models for patch spraying purposes (e.g. Paice et al., 1998). In this regard, our model could be improved by modelling interspecific competition mechanistically. In order to simplify the model we have divided the soil into two layers: a shallow layer from which seeds can germinate and a deep layer. However, in reality the soil is a continuum and there will be a gradient over which seeds can germinate at different rates. Finally, natural seed dispersal is barochorous in our model and seeds are moved by the combine and cultivator. Both of these methods of dispersal are independent of other factors, yet it has been shown that there can be some influence of wind speed on seed dispersal of *A. myosuroides* (Colbach and Sache, 2001) and equally, seed movement in the soil can depend on soil properties (Benvenuti, 2007).

In our model validation, the large scale correlations between *A. myosuroides* and soil properties were generally similar to those observed in the field. However, for soil pH our simulations predicted positive correlations at large scales, whereas the observed data showed a negative correlation. The only role of soil pH in our model is in altering the asymptote reached in germination. We implemented this using a threshold approach, where the asymptote for germination is increased when soil pH is below 6.5. It is possible that this is insufficient to describe the true nature of the impact of soil pH on germination as by only changing the asymptote we will only see these differences in years when there are very large numbers of seeds germinating. It is likely that the observed response to pH in the field data is a product of additional processes to do with growth and competition in the established phase that are not currently captured in the model.

The usefulness of our model in its ability to predict patch locations for *A. myosuroides* highlights the possibility of using similar models for other species where data are available on the response of the species to various soil properties. This model could be used for other grass weeds with similar life-cycles and the key aspects of the life-cycle altered to fit with known responses of that species to environmental properties. This would allow the prediction of patches of problematic weed species based on soil maps and could be used in conjunction with current patch mapping activities to zone fields for site-specific weed management at the appropriate scale.

## 7 Conclusions

We have drawn together experimental data on the impact of soil properties on the life-cycle and management of this important agricultural species and through a modelling approach demonstrated the important role played by soil properties in determining the within-field distribution of *A. myosuroides*. We have also shown that scale-dependent correlations between *A. myosuroides* and soil properties observed in the field are an emergent property of this model, which incorporates small changes to individual components of the life-cycle due to soil properties. This could allow it to become an effective management tool as the coarse-scale correlations, which are shown to be of the greatest importance, are the ones that have the most relevance to management.
