# Supplementary material for: Modelling the effect of spatially variable soil properties on the distribution of weeds
Source: Ecol Modell. 2019 Mar 24;396:1–11. doi: 10.1016/j.ecolmodel.2018.11.002 (PMC6472614; doi:10.1016/j.ecolmodel.2018.11.002)
Supplement: Supplementary file 1 [file mmc1.pdf]

Table S.1. The volumetric composition of the three soil mixtures used in Experiment 1, their percentage organic matter measured by loss on ignition, and the measured pH of the soils.

Soil mixture	Coarse sand (% by volume)	Fine sand (% by volume)	Loam (% by volume)	Composted bark (% by volume)	OM (LOI % w/w)	pH
Low organic matter	35	35	22.5	7.5	1.93	7.16
Medium organic matter	20	20	45	15	2.37	7.00
High organic matter	0	0	75	25	6.15	7.00

heads produced by the *A. myosuroides* plant in each pot.

S.2. Calculation of parameters used for germination (Equation 3)

M, a, c and x_{50} in equation 3 are determined by properties relating to the seeds including the age of the seed (days, A), time from germination to maturity of the mother plants (days, t_{GM}), water deficit between flowering and maturity (mm, W_{def}), depth of seed (cm, D), hydrothermal time spent in darkness prior to tillage (t_{DH}), mean seed mass (g, M_{S}), and total available nitrogen (kg ha^{-1}, N).

M, the maximum level of germination is given by

$$M = M_0 \, \frac{0.5311 - 0.00947\, D}{0.5311} \exp(-0.00115\, t_{\mathrm{DH}}^{1.121}) \tag{18}$$

where

$$\begin{aligned} M_0 = {} & 0.924 - 0.000149\, t_{\mathrm{GM}} + 0.391 \mathrm{e}^{-0.033\, A} \\ & - 0.00380\, t_{\mathrm{GM}}\, \mathrm{e}^{-0.033\, A} + 0.00077\, W_{\mathrm{def}}\,. \end{aligned} \tag{19}$$

The parameter a, which is the lag phase of germination, is given by

$$a = 0.95664\, \frac{1}{v_m}\, a_0 \tag{20}$$

where

$$\begin{aligned} a_0 = {} & 49.78 - 66.43\, \mathrm{e}^{-0.0086\, A} - 0.0022\, t_{\mathrm{GM}} \\ & + 0.358\, t_{\mathrm{GM}} \cdot \mathrm{e}^{-0.0086\, A} \end{aligned} \tag{21}$$

and

$$v_m = \frac{0.5311 - 0.00947\, D}{0.5311}\,. \tag{22}$$

The shape parameter c is given by

$$c = 0.95664\, v_m\, c_0 \tag{23}$$

where

$$c_0 = 0.125 - 1.997\,\mathrm{e}^{-0.063A} + 0.00676\, t_{\mathrm{GM}} + 0.0199\, t_{\mathrm{GM}}\,\mathrm{e}^{-0.063A} + 0.0101\, W_{\mathrm{def}} + 246.9\, M_{\mathrm{S}} - 0.00702\, N \tag{24}$$

and the time to 50% germination, x_{50}, is given by

$$x_{50} = \frac{1.04533}{v_m}\, x_0 \left(\mathrm{e}^{0.212 t_{\mathrm{DH}}^{1.121}} - 1\right) \tag{25}$$

where

$$x_0 = 65.72 + 200.99\,\mathrm{e}^{-0.044A} + 0.0968\, t_{\mathrm{GM}} - 1.086\, W_{\mathrm{def}} \tag{26}$$

S.3. Derivation of relationship between soil organic matter and head production

We compared the average number of heads per plant for each treatment to the average value across the whole experiment to give a scaling factor. We then scaled the value produced by Equation 10 for one plant by this value to give the expected number of heads for each treatment under field conditions. We then parameterised the equation for each of our experimental soil types. We had no reason to assume that the asymptote might change with soil and so we kept this constant at

$$\text{asymptote} = \frac{\beta}{\alpha} = 1517.157 \tag{27}$$

We then rearranged to find α and β by substituting in the number of heads when there is one plant (H_1)

$$\alpha = \frac{H_1}{1517.157 - H_1} \tag{28}$$

$$\beta = 1517.157\,\alpha \tag{29}$$

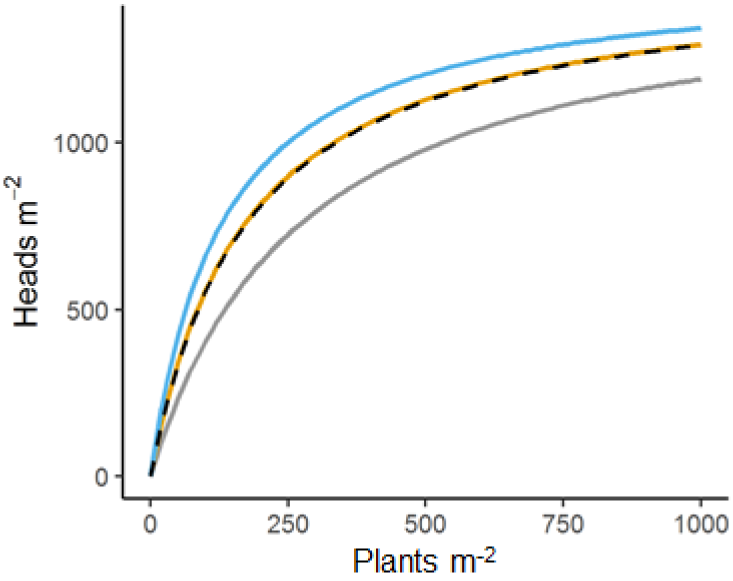

Figure S.1. Density dependent relationship between plants and heads (m^{-2}) with curve parameters β and α adjusted according to soil organic matter. The relationship and parameterisation described by Moss *et al.* (2010) is shown by a dashed black line, other curves show parameterisations for experimental data: grey is soil low organic matter; yellow is medium soil organic matter and blue is high soil organic matter. See supplementary material for experimental detail.

When we parameterised the curve in this manner for the scaled mean value at one plant for each of the three soil properties in our experiment we got different curves for each organic matter all reaching the same asymptote but with different slopes (Figure S.1). On the highest organic matter the number of heads increased rapidly with the number of plants, whereas on low organic matter the number of heads increased more steadily as plant numbers increase. On the medium organic matter, we saw a response very like that observed by Moss *et al.* (2010), for which they provided the original parameterisation of the equation.

To make this relationship more general across a range of soil organic matter

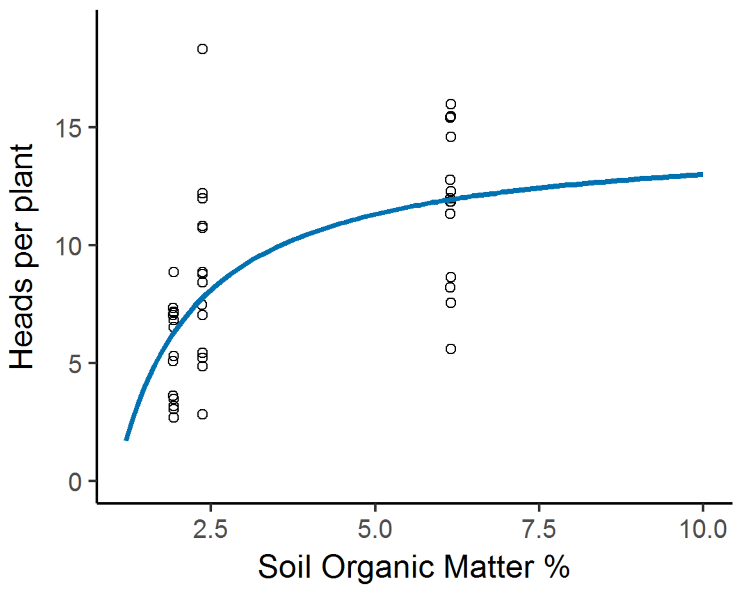

Figure S.2. Relationship between soil organic matter and the number of heads per plant. The black discs are experimental data and the blue line is the curve fitted is from Equation 30. See supplementary material for experimental detail.

contents we plotted all the original data values for the number of heads per plant at the three levels of organic matter (Figure S.2) and fitted a curve

$$H_1 = \frac{\xi\, S_{\text{SOM}}}{1 + \omega\, S_{\text{SOM}}} + \varsigma \tag{30}$$

where S_{SOM} is the percentage soil organic matter, and ξ, ω, and ς are the parameters fitted to the data. The fitted values were $\xi = 844.4883$, $\omega = 6.9542$, and $\varsigma = -106.7242$ (Figure S.2).

We used this relationship to find α and β (Equations 28 and 29) and then used Equation 10 to determine the number of heads produced.

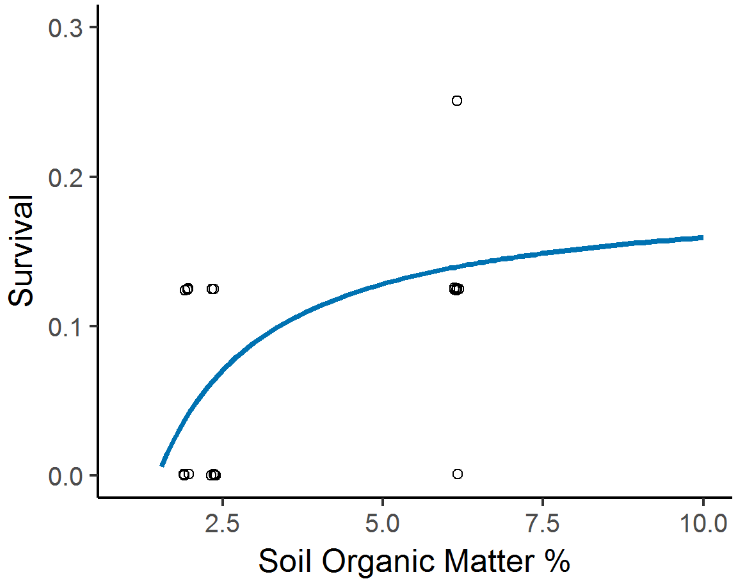

Figure S.3. Relationship between soil organic matter and survival after the application of pre-emergence herbicide. The discs are data from Metcalfe *et al.* (2018a) and the blue fitted curve is the optimised form of Equation 9.

S.3.0.1. Relationship between soil organic matter and survival after the application of pre-emergence herbicide.

S.4. Supplementary References

Colbach N, Chauvel B, Dürr C, Richard G, 2002a. Effect of environmental conditions on *Alopecurus myosuroides* germination. I. Effect of temperature and light. *Weed Research*, **42** (3) 210–221.

Colbach N, Dürr C, Chauvel B, Richard G, 2002b. Effect of environmental conditions on *Alopecurus myosuroides* germination. II. Effect of moisture conditions and storage length. *Weed Research*, **42** (3) 222–230.

Moss SR, Storkey J, Cussans JW, Perryman SAM, Hewitt MV, 2004. The Broadbalk long-term experiment at Rothamsted: what has it told us about weeds? *Weed Science*, **52** 864–873.

Moss SR, Tatnell LV, Hull R, Clarke JH, Wynn S, Marshall R, 2010. *Integrated management of herbicide resistance.* HGCA Project Report **466**.
